# Fabrication and Characterisation of MWCNT/Polyvinyl (PVC) Polymer Inclusion Membrane for Zinc (II) Ion Removal from Aqueous Solution

**DOI:** 10.3390/membranes12101020

**Published:** 2022-10-20

**Authors:** Nadia Aqilah Khalid, Noor Fazliani Shoparwe, Abdul Hafidz Yusoff, Ahmad Ziad Sulaiman, Abdul Latif Ahmad, Nur Aina Azmi

**Affiliations:** 1 Gold Rare Earth and Material Technopreneurship Centre, Faculty of Bioengineering and Technology, Jeli Campus, Universiti Malaysia Kelantan, Jeli, Kota Bharu 17600, Kelantan, Malaysia; 2 School of Chemical Engineering, Engineering Campus, Universiti Sains Malaysia, Nibong Tebal 14300, Pulau Pinang, Malaysia; 3 Benua Sunda Cari Gali Sdn Bhd. No 6, Medan Pusat Bandar 1, Seksyen 9, Bandar Baru, Bangi 43650, Selangor, Malaysia

**Keywords:** polymer inclusion membrane, multiwalled-carbon nanotubes, bis-(2-ethylhexyl) phosphate, zinc (II) ions, extraction, adsorption

## Abstract

Heavy metal pollution has prompted researchers to establish the most effective method to tackle the impacts of heavy metals on living things and the environment, which include by applying nanoparticles. An example is the employment of multi-walled carbon nanotubes (MWCNTs) as an additive in an intermediate membrane or polymer inclusion membrane (PIM). The MWCNTs were added to enhance the properties and reinforce the transport performance of zinc (II) ion (Zn^2+^) removal from the source phase to the receiver phase by the PIMs. The present study constructed a membrane with a poly(vinyl chloride) (PVC)-based polymer, dioctyl phthalate (DOP) plasticiser, and bis-(2-ethylhexyl) phosphate (B2EHP) carrier incorporated with different concentrations of MWCNTs. The contact angle (CA), water uptake, ion exchange capacity (IEC), and porosity of the fabricated membranes were evaluated. The membrane was also characterised by employing scanning electron microscopy (SEM), Fourier transform infrared spectroscopy (FTIR), differential scanning calorimetry (DSC), and electrochemical impedance spectroscopy (EIS). Subsequently, the fabricated PIM (W1) and mixed matrix (MM)-PIM (W2–W5) samples were assessed under different parameters to acquire the ideal membrane composition and effectiveness. Kinetic modelling of Zn^2+^ removal by the fabricated PIMs under similar conditions was performed to reveal the mechanisms involved. The average removal efficiency of the membranes was >99% at different parameter conditions. Nevertheless, the W3 membrane with 1.0 wt% MWCNT immersed in a 5 mg/L initial Zn^2+^ concentration and 1.0 M receiver solution for seven hours at pH 2 demonstrated the highest percentage of Zn^2+^ removal. The experimental data were best fitted to the pseudo-first-order kinetic model (PFO) in kinetic modelling, and the permeability and flux of the W3 at optimum conditions were 0.053 m s^−1^ and 0.0532 mol m^−2^ s^−1^, respectively. In conclusion, the transport mechanism of Zn^2+^ was enhanced with the addition of the MWCNTs.

## 1. Introduction

Over the years, diverse methods that emphasise the experimental analysis of heavy metal removal have been researched. Heavy metals, such as copper (Cu), cobalt (Co), manganese (Mn), iron (Fe), and zinc (Zn), are generally identified as a subclass of constituents with metallic characteristics [1]. Long-term excessive exposure to heavy metal ions might lead to toxin accumulation in the body, which is a major health concern. 

Researchers have investigated various methods to overcome the growth in heavy metal pollution problems from e-waste. The treatment methods are classified into chemical, physical, and biological treatment. Physical and chemical treatments are costly as compared to biological treatment. Although researchers use renewable sources in adsorption methods to save costs, such as biomass waste in Granular Activated Carbon, the whole production process is expensive and involves complex operations [2]. On the other hand, biological treatment is labour- and time-consuming, including biosorption to remove heavy metals using *Ulva lactuca* algae-based chitosan bio-composites from processed shrimp shells [3]. This has diverted interest toward focusing on a new membrane technology known as the polymer inclusion membrane (PIM), a type of liquid membrane (LM). 

The PIMs permit co-current extractions and back-extractions at opposite membrane phases [4,5]. The key to successful PIM fabrication relies on the membrane formulation, consisting of a base polymer, carrier, and plasticiser [6]. The standard base polymers utilised in the formulation of PIMs, such as cellulose triacetate (CTA) and poly (vinyl chloride) (PVC), which offer the mechanical resilience of membranes, are summarised in Table 1. A PVC polymer is more repellent to acidic solutions than a CTA polymer, which preserves the membrane [7]. A carrier is a complexing agent or ion exchanger that binds target ions and permeates the membrane [8]. Some examples are di-(2-ethylhexyl) phosphoric acid (D2EHPA), bis-(2-ethylhexyl) phosphate acid (B2EHPA), and acetylacetone (ACAC). Plasticisers are organic compounds that act as membrane supplementation to develop membrane fluidity, flexibility, and softness and enhance the consistency of the components [7,9,10]. However, the inclusion of a plasticiser in PIMs is optional. This is owing to the fact that some extractants also have characteristics in common with plasticisers [11], such as p-nitrophenyl pentyl ether (2-NPPE), *o*-nitrophenyl octyl ether (2-NPOE), poly(butylene adipate-co-terephthalate) (PBAT), dioctyl phthalate (DOP), bis (2-ethylhexyl) adipate (DAO), adenosine (ADO), and phosphonium ionic liquids (ILPs). The development of optimal PIMs for heavy metal removal, particularly Zn^2+^, was investigated in the present study.

Several studies have recently revealed that the major downside of PIMs is sustaining the longstanding constancy of reused membranes due to membrane components’ loss (extractant and plasticiser) into the aqueous solution in the long term [19,20,21]. The disadvantage is attributable to the hydrophobic properties of the membranes, which generate membrane fouling due to undesirable solute build-up [22]. Studies have suggested that nanofillers strengthen the membrane polymeric matrix in terms of chemical mechanical and thermal uniformity in harsh conditions, as well as enhancing the separation properties of the membranes due to the combinatorial features of the organic–inorganic compounds [23,24,25,26,27,28,29]. For example, various inorganic nanoparticle-doped (ferrite, (Fe_3_O_4_)), commercially available TiO_2_ and SiO_2_, and multi-walled carbon nanotubes (MWCNTs)) PIMs incorporated with Aliquat 336 were employed in the removal of arsenate and phosphate [4]. Nonetheless, no studies on the utilisation of MWCNT-based PIMs configured to remove zinc (II) ions from aqueous solutions have been explored to date. Consequently, Zn^2+^ removal with a fabricated heterogeneous mixed matrix (MM) polymer membrane by incorporating MWCNT nanoparticles in PVC-based PIMs was conducted in the present study.

The MWCNTs have attracted attention due to their superior properties that make them excellent support materials, such as great stability in acidic conditions, large specific surface area, strong interactions, and no swelling [30]. Furthermore, the nanotubes have a highly advanced hydrophobic surface with strong sorption properties across various compounds, establishing outstanding adsorption capability to remove toxic ions from wastewater [31,32]. Studies have suggested that involving MWCNTs as potential adsorbents in membrane-based separation accelerates the transport of target ions against a concentration gradient in the feeding phase towards the surfaces of PIMs to bind with extracting agents under an ionic exchange process prior to back-extraction into the receiving phase [4,33]. Furthermore, the desorption process undertaken by the MWCNTs might co-occur at the receiving interphase to facilitate the recovery of the target ions and reduce the fouling accumulation [34]. Consequently, the kinetic models of Zn^2+^ were examined to determine the mechanism and performance of the process.

The effects of MWCNT load on the physical and chemical properties of the fabricated PIMs in the present study were studied in terms of hydrophobicity, porosity, surface morphology, functional groups, water absorption, ion exchange capacity (IEC), temperature difference, and conductivity. The current study synthesised MWCNTs into optimum PIMs that were composed of PVC as the base polymer, bis-(2-ethylhexyl) phosphate (B2EHP) as the carrier, and dioctyl phthalate (DOP) as the plasticiser, to remove Zn^2+^ from aqueous solutions. Accordingly, the current study would provide better knowledge of the efficiency of MM MWCNT-incorporated PVC-PIMs (MM MWCNT/PVC-PIMs) in removing Zn^2+^.

## 2. Materials and Methods

### 2.1. Materials

The PVC, B2EHP, dioctyl phthalate (DOP), tetrahydrofuran (THF), and zinc nitrate were supplied by Sigma-Aldrich (St Louis, MO, USA). The 38% hydrochloric acid (HCl), 65% nitric acid (HNO_3_), and phenolphthalein were supplied by HmbG^®^ Chemicals (Hamburg, Germany). Sodium hydroxide (NaOH) and sodium chloride (NaCl) were supplied by R&M Chemicals (Selangor, Malaysia), while Nanotech provided the MWCNTs.

### 2.2. Fabrication of the MWCNT/PVC-PIMs

The MM MWCNT/PVC-PIMs were fabricated by employing the dry phase inversion technique [35]. The compositions of the doped solutions utilised are listed in Table 2. The casting solution was prepared by mixing a predetermined PVC powder with a THF solvent before adding B2EHP and DOP solutions. The resultant solution was continuously stirred for four hours at 400 rpm with a stirring hotplate at 60 °C to disintegrate the cluster particles, until a clear and homogenous solution was acquired. Sonication treatment was applied on the doped solution when higher MCNT content was mixed in order to enhance its distribution. Each polymeric solution was placed on a casting machine with a blade thickness of 0.2 mm. The casted membrane was left overnight to dry, before peeling off the casting glass and cutting into the required membrane shape. The membrane was washed a few times with distilled water to remove excess solvent and stored for further analysis. In the current study, the fabricated PIM was denoted as W1 and the mixed matrix (MM)-PIM samples were represented as W2–W5.

### 2.3. Characterisation of the Fabricated PIMs

#### 2.3.1. The Scanning Electron Microscopy (SEM) Analysis

The membrane was cut to 5 mm × 5 mm pieces and coated with gold prior to observing the surface morphology with SEM (JEOL, Tokyo, Japan, JSM-IT100). The surface morphology of the membranes was viewed under 100× magnification and a 10 kV acceleration voltage [12].

#### 2.3.2. The Contact Angle (CA) Study

The current study employed a 250-U1 contact angle (CA) goniometer by Ramehart Instrument (Succasunna, NJ, USA). The contact angle determination was performed on the PIMs at room temperature. The sessile drop technique utilised distilled water, which was dropped onto the membrane surface to identify the wettability of the fabricated PIMs. Following the deposition of water droplets on the surfaces of dried PIMs, the CA readings were taken, and an image of the distilled water dropped was captured with a digital camera.

#### 2.3.3. The Water Uptake Analysis

Water uptake evaluations were conducted to measure the amount of water absorbed by the membranes. Dry PIMs were immersed in distilled water for 24 h after their weights were established. After 24 h, the PIMs were removed from the water and rubbed between tissue papers to remove water deposited on the membrane surface prior to being weighed. The average water uptake values of three membrane samples were taken. The water uptake of the samples was derived by employing Equation (1) [36].

(1) $$\mathrm{Water}\;\mathrm{uptake}=\frac{W_{wet}-W_{dry}}{W_{dry}}\times 100\%$$

where W*_wet_* is the weight of wet membranes and W*_dry_* is the weight of dried membranes.

#### 2.3.4. The Porosity Evaluation

A basic gravimetric technique was utilised to measure the overall porosity of the fabricated PIMs. The membrane porosity (ε) could be defined as the total volume of the membrane divided by the volume of pores. The 2.0 × 2.0 cm^2^ membrane samples were immersed in distilled water for 48 h. Subsequently, the membranes were removed and wiped between filter papers before being swiftly measured and oven-dried at 25 °C for over 10 h. Finally, the dried membranes were weighed once more to obtain the final weights of membranes. The tabulated weights were estimated with Equation (2) [37].

(2) $$\varepsilon \left(\%\right)=\frac{\left[W_{w}-W_{d}\right]/\rho_{w}]}{\left[\left(W_{w}-W_{d}\right)/\rho_{w}\right]+\left(\frac{\left[W_{d}\right]}{\rho_{p}}\right)}\times 100$$

where W_w_ is the weight of wet membranes (g), W_d_ is the weight of dried membranes (g), *ρ_W_* is the pure water density at working condition (g cm^−3^), and *ρ_p_* is the polymer density (g cm^−3^).

#### 2.3.5. The Fourier Transform Infrared Spectroscopy (FTIR) Analysis

The functional groups of the components present in the fabricated samples were evaluated with FTIR. The results were compared with existing functional groups’ bond standards, such as alkyl halides (–C–F, –CF_2_, and –CF_3_), aromatic groups, alkanes (–CH and –CH_2_), esters, alcohols (–P–OH), carbonyl groups (C=O), carbon–carbon (C–C and C–C–C), and hydroxyl groups (O–H) [38,39,40]. The FTIR was conducted with a iZ10 FTIR Spectrometer (Thermo Fisher Scientific, Waltham, MA, USA). The spectrum recorded was between the 400 and 4000 cm^−^^1^ wavenumber at 16 scans and a 4 cm^−^^1^ resolution. The samples were tested with the transmission method and the spectra were analysed with the OMNIC software (Thermo Fisher Scientific, Waltham, MA, USA) [41].

#### 2.3.6. The Differential Scanning Calorimeter (DSC) Analysis

The DSC was performed to investigate the thermal behaviours of the fabricated PIMs with varying nanotube loads [23]. The analysis was conducted with the STA 8000 from PerkinElmer, Waltham, MA, USA. Approximately two milligrams of each membrane sample were analysed in a nitrogen atmosphere at 30 to 600 °C and a 10 °C/min heating rate. The results were analysed with the STARe Evaluation software (Mettler, Toledo, Shah Alam, Malaysia).

#### 2.3.7. The IEC Evaluation

The present study measured the IEC of the fabricated membranes via the titration method. The membrane was cut to 2 cm × 2 cm samples and soaked in 1 mol/dm^3^ HCl for 24 h, removed, and rinsed with distilled water to remove excess HCl on the surface of the membrane. The membrane was then submerged in 1.0 mol/dm^3^ NaCl solution for 24 h. Finally, the samples were removed and the remaining solution was titrated with 0.01 mol/dm^3^ NaOH solution that contained a few drops of phenolphthalein as the indicator [42]. The IEC of the membrane was determined with Equation (3).

(3) $$\mathrm{IEC}=\frac{\mathrm{ab}}{W_{\mathrm{dry}}}$$

where a is the concentration of the titrated NaOH solution (mol/dm^3^), b is the volume of the NaoH solution (dm^3^), and W_dry_ is the weight of the dry membrane sample (g).

#### 2.3.8. The Electrochemical Impedance Spectroscopy (EIS) Analysis

The membranes were cut into 4 cm × 4 cm pieces before being sandwiched in a coin-cell system and connected to an EIS potentiostat CS23 from Corrtest Instruments, Wuhan, China. The present study employed a 10 mV amplitude and a frequency between 10 μHz and 10 MHz. The measurements were assessed with an open-circuit potential (OCP) and analysed with the CS Studio 5 software to identify the bulk resistances, *R_b_*. The ion conductivity, σ, of the membrane samples was calculated according to Equation (4) [43].

(4) $$\sigma =\frac{d}{\left(R_{b\;}\times S\right)}$$

where *d* is the thickness and *S* is the surface area of the membrane samples.

### 2.4. The H-Cell Apparatus Arrangement

**Figure 1:**
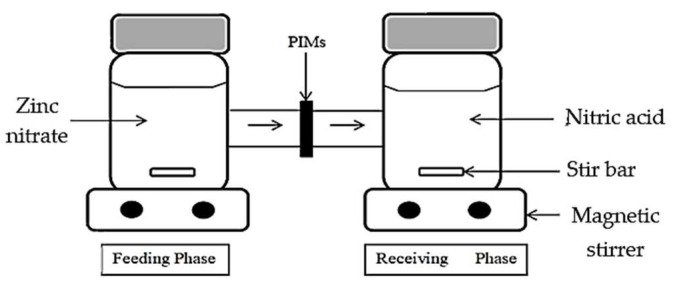
The H-cell device setup.

The Zn^2+^ removal was conducted by employing an H-cell apparatus, where the analytes were extracted from a stripping phase into a receiving phase by diffusion through a polymer film. The apparatus was divided into two compartments and a fabricated membrane sample was clamped between the feeding and receiving phases. The zinc nitrate solution was inserted in the feeding phase, while 0.1 M nitric acid was the receiving phase. The solutions were agitated continuously to acquire uniform conditions during the experiment. The arrangement of the H-cell apparatus is displayed in Figure 1.

### 2.5. The Zn^2+^ Removal Performance Studies

A performance study was conducted in the H-cell apparatus by employing 150 mL of 10 mg/L zinc nitrate solution as the feeding phase. During the Zn^2+^ removal process, the feeding and receiving phase solutions were continuously stirred for five hours with magnetic stirrers at 350 rpm. The pH of the aqueous solution was modified to 6.0 ± 0.2 with 1.0 M HCl and 1.0 M NaOH. Samples (10 mL) from both compartments were collected every hour to determine the concentration of Zn^2+^ through atomic absorption spectroscopy (AAS).

A PinAAcle 900F (Perkin Elmer, Waltham, MA, USA) was employed to perform the AAS analysis. The steps were repeated by varying the fabricated PIMs. Based on the results, the ideal mixed matrix membrane was further examined under different parameters, namely different initial zinc nitrate (5, 10, 20, 30, 40, and 50 mg/L) and receiving phase (0.1, 0.5, 1.0, 1.5, and 2.0 M nitric acid) concentrations. The removal efficiency percentage (E%) was calculated with Equation (5).

(5) $$E\%=\frac{{\left(\mathrm{zinc}\right)}_{i}-{\left(\mathrm{zinc}\right)}_{f}}{{\left(\mathrm{zinc}\right)}_{i}}\times 100\%$$

where (zinc)_i_ is the initial zinc nitrate concentration in the aqueous phase (mg/L) and (zinc)_f_ is the final zinc concentration after the removal in the aqueous phase (mg/L).

### 2.6. The Kinetic Studies

Removing Zn^2+^ is a straightforward process but involves a complex mechanism, requiring a kinetic modelling study to describe the nature and process of the technology. The reaction rate (K_1_) of the predicted removal profile was obtained with the polymath Fogler software version 6.10 through a non-linear least square regression analysis [44]. The pseudo-first-order (PFO) and pseudo-second-order (PSO) models, respectively, represented by Equations (6) and (7), were proposed as the kinetic models. The data were then fitted with the theoretical data presented by the software.

(6) $$C=C_{e}-\exp\left(-k_{1}t\right)\left(C_{e}-C_{0}\right)\;$$

where *k*_1_ is the rate constant of extraction (g/(mg min)), *C_e_* is the equilibrium extraction capacity, and $C_{0}$ is the amount of Zn^2+^ removed (mg/g).

(7) $$C=C_{e}+\frac{1}{k_{2}t-\frac{1}{C_{e}-C_{0}}}\;\;$$

where *k*_2_ is the rate constant of extraction (g/(mg min)), *C_e_* is the equilibrium extraction capacity, and $C_{0}$ is the amount of Zn^2+^ removed (mg/g).

### 2.7. Permeability and Flux Assessments

The permeability, P, and flux, J, of Zn^2+^ transport were first calculated based on the kinetics of the first-order reaction of the mechanism according to Equation (8) [45]. First-order reaction kinetics signified a linear correlation produced between ln(*C*/*C_i_*) and *t*, supplying values in high determination coefficient (r^2^) ranges. The data were then inserted into Equation (9) to obtain the permeability coefficient (*P*). Lastly, the initial flux (*J_i_*) of the transport process was determined with Equation (10).

(8) $$\ln\frac{C}{C_{i}}=-kt$$

(9) $$P=\frac{V}{A}k$$

(10) $$J_{i}=\;P\cdot C_{i}$$

where *V* is the volume of the aqueous solution in the stripping phase and *A* is the area of the polymer membrane.

## 3. Results and Discussion

### 3.1. The SEM Analysis

**Figure 2:**
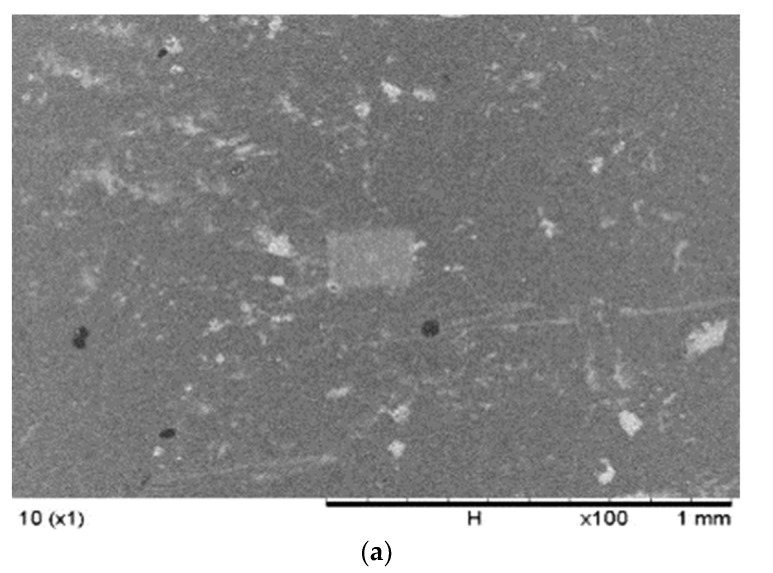

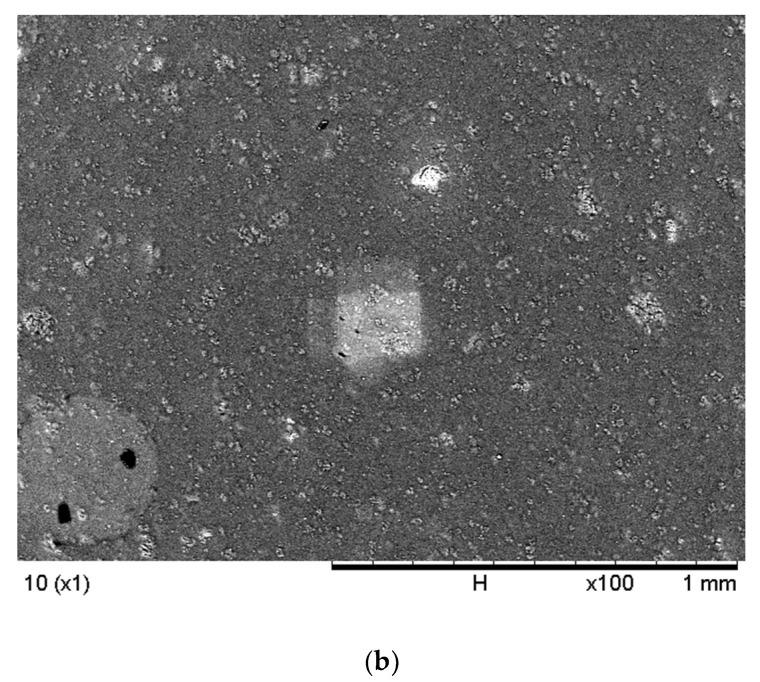
The SEM images of the (**a**) PIM (without nanoparticles) and (**b**) MM-PIM (with nanoparticles).

Figure 2 demonstrates the comparison of the surface morphologies of the membranes with and without nanoparticles. The SEM analysis was conducted at 100× magnification to evaluate the morphology of the microstructure on the surfaces of the fabricated membranes formed from the insertion and distribution of carriers and nanoparticles in the membrane polymer matrix. Apparent changes were observed in the morphology of the membranes with the addition of nanoparticles. The PIM sample in Figure 2 presented a smooth surface with fewer microspores, possibly due to the effects of plasticisers, which smoothen polymer surfaces and enhance the mechanical strength of the membrane. Moreover, [46] reported that polymers incorporated with plasticisers demonstrated reduced crystalline properties and an improved amorphous nature, which are essential to developing the ionic conductivity of the membranes.

The MM-PIMs had denser and coarser surfaces, although the added plasticiser capacity was equivalent to the PIMs without nanoparticles. The observation indicated that the pores on the membrane surface filled with carbon nanotubes, resulting in a slightly rough surface with evidently less agglomeration of MWCNTs [47]. A similar result was reported by [48], who employed reduced graphene oxide (rGO) nanoparticles in a PIM. A favourable fibrous structure was found on the surface morphology of the membrane polymer matrix after the loading the rGO nanoparticles into the PIM. The finding exhibited the good dispersion of organic fillers into the solution of the casting membrane.

No visible defects or cracks were observed on the MM-PIM membrane surfaces, implying no unfavourable impact on the stability of the membrane with the insertion of MWCNTs. Nonetheless, [49] reported that the amount of inorganic filler utilised was crucial to the morphology of the fabricated membranes as it could significantly damage the stability of the membrane, which would lower the ability of the membranes to transport analytes. The study implied that the nanoparticle compositions in membrane casting solutions could heavily influence the stability and membrane efficiency.

### 3.2. The FTIR Analysis

**Figure 3:**
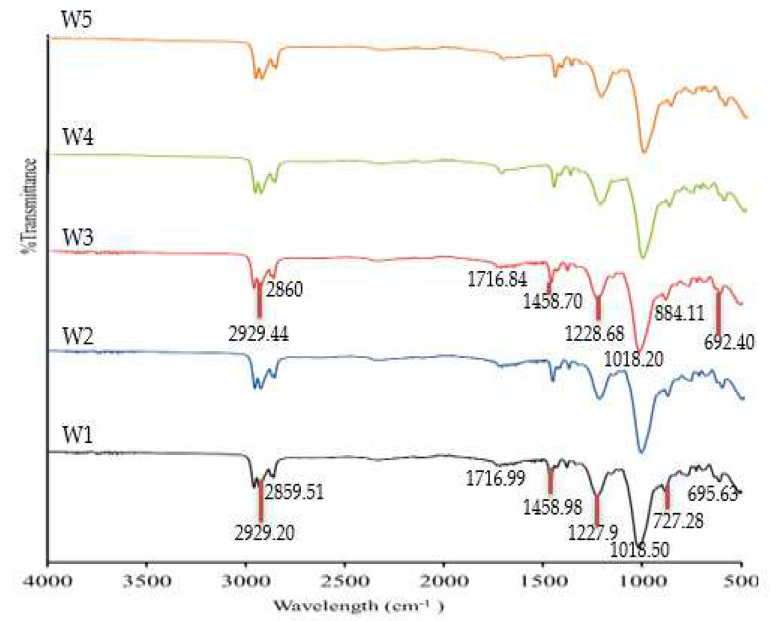
The FTIR spectra of the fabricated membranes prior to Zn^2+^ removal.

**Figure 4:**
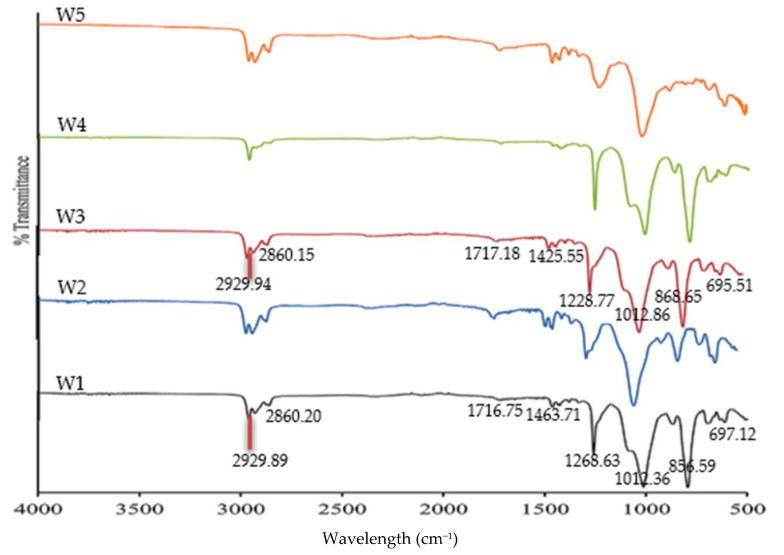
The FTIR spectra of the fabricated membranes after Zn^2+^ removal.

The components of the PIMs manufactured in the current study were evaluated with FTIR. The FTIR spectra of the W1, W2, W3, W4, and W5 samples before and after Zn^2+^ removal are illustrated in Figure 3 and Figure 4. The identified molecular vibrations observed on the W1 membrane (PVC 18%, B2EHP 30%, DOP 1%, and THF 51%) and W3 membrane (MWCNT 1%, PVC 18%, B2EHP 30%, DOP 1%, and THF 51%) before and after Zn^2+^ removal are summarised in Table 3. By observing the FTIR spectra in Figure 3, we can find that there were no apparent wavelength differences discerned between the W1 membrane and the MM-PIMs before Zn^2+^ removal. Despite slight dissimilarities between the W1 and W3 wavenumbers, the spectra were placed in similar functional groups.

Two weak peaks indicated the presence of asymmetric methyl and symmetric methylene stretching from B2EHP, DOP, and MWCNT components. A very weak wavelength appeared within the 1716.84–1716.99 cm^−1^ range, demonstrating a carbonyl group bending, while the peaks within the 1458.70–1458.98 cm^−1^, 1227.90–1228.68 cm^−1^, and 884.73–727.0 cm^−1^ ranges corresponded to C–C stretching, O–C bending, and C–H groups, respectively, from the DOP component. Broad and sharp peaks were revealed at 1227.90–1228.68 cm^−1^ and 1018.20–1018.50 cm^−1^, attributable to the P=O stretching and P–O group, respectively, from the B2EHP component. The PVC component was also distinguished on a weak peak within the 692.40–695.63 cm^−1^ range corresponding to C–Cl stretching. The findings indicated that incorporating MWCNTs into the PIMs did not significantly affect the MM-PIMs’ spectra and was not entirely responsible for the presence of the functional groups in the membrane.

The FTIR spectra of the W3 membrane before and after Zn^2+^ removal were compared. Apparent wavelength shifts post-Zn^2+^ removal process were observed on the W3 MM-PIM spectra. Figure 4 demonstrates a minor intensity decrement at bands 2929.94 and 2860.15 cm^−1^ of the W3 membrane after Zn^2+^ removal, indicating the cleaving of alkyl chains from the surfaces of the carbon nanotubes [25]. A medium and sharp band at 1258.77 cm^−1^ attributable to the symmetrical phosphate in B2EHP and the carbonyl group in the DOP structure was detected. The results suggested that the extraction of ions delivered less effect on the intensity of the component. Alternatively, notably reduced wavelength intensity emerged after the extraction process on the intense and sharp peak at 1012.86 cm^−1^ (P=O stretching) and the broad peak at 868.65 cm^−1^ (C–H bending). The results suggested that the interaction between the carrier components and the polymer diffused the Zn^2+^ through the MM-PIMs.

### 3.3. The CA, Porosity, Water Uptake, and Thickness Evaluations

The CA was conducted to investigate the hydrophobicity of the membrane samples. A hydrophobic membrane possesses a greater CA (>90°), whereas a hydrophilic membrane is considered to have a lower CA (<90°) [50,51]. The CA, porosity, water uptake, and thickness of the samples are listed in Table 4. Porosity, water uptake, and thickness corresponded to each other, and, from the results, the MWCNTs influenced the hydrophilicity of the membranes. As the percentage of the nanomaterial increased, the water CA on the membrane surface decreased. The W1 PIM had a CA of 52.5°, which declined to the smallest CA of 42.5° with a 1.5% MWCNT load. The observations revealed that the inclusion of MWCNTs reduced the membrane’s CA and improved its hydrophilicity. Nevertheless, as the MWCNT content was increased to 2.0%, the membrane’s hydrophobicity escalated slightly to 43.9°. The main factor that might have lowered the membrane’s wettability was the uneven distribution or irregular dissemination of the carbon nanotubes on the PIMs. The irregular distribution was impacted by the intense van der Waals bond strength, leading to aggregation at higher concentrations of MWCNTs [52,53].

Contrary to the CA measurements that demonstrated the hydrophilic characteristics of the nanotubes, the fabricated MM-PIMs exhibited a declining trend of water uptake compared to the PIMs without nanoparticles (W1) (see Table 4). Initially, the W1 membrane yielded a *U* value of 50.21%, which was reduced to 39.25% after the incorporation of MWCNTs within 0.5 to 2.0%. The reason might be the core of physical correlation within the molecules in the polymer network, which obstructed the fluidity of the polymer chain, leading to alleviated water intake capacity [54].

Despite containing the highest MWCNT load, the W5 membrane exhibited the largest aggregation of carbon tubes and a smaller interface area between the polymer matrix and the nanomaterial; however, the sample demonstrated the lowest water uptake percentage. The result was most likely due to the aggregation of the nanoparticles, which covered the membrane surface and blocked the water from flowing inside the membrane, creating a pore-clogging phenomenon as the carbon nanotubes were poorly distributed on the PIMs’ surface [55,56].

The overall porosity percentage, ε, of the membranes declined with an increased nanoparticle concentration. A higher carbon nanotube load (>1.5%) formed membranes with denser structures and lower porosity due to higher casting solution viscosities [57], resulting in a greater propensity of nanomaterials to aggregate, which likely obstructed the membrane pores. A study by [22] reported that membranes with lower porosity tended to generate a proportionately lower water flux due to the increasing concentration of the nanomaterials in the nanocomposite membranes. The findings implied that the aggregation of nanomaterials in the membranes could reduce the porosity, which minimises the mobility of ions transferred through the PIMs, reducing the transportation efficiency [58].

### 3.4. The DSC Analysis

**Figure 5:**
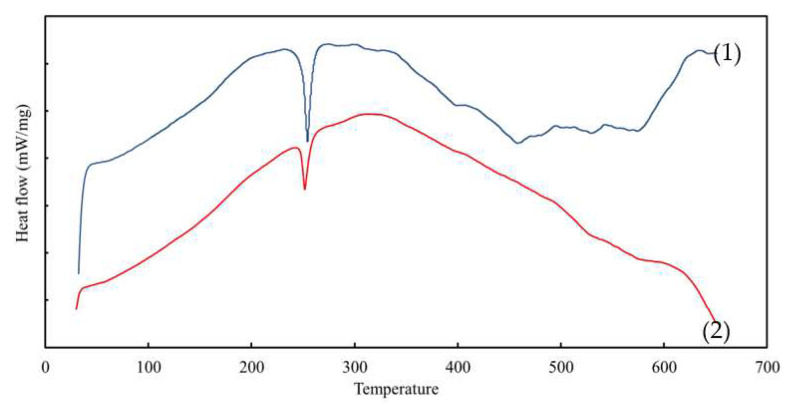
The DSC curves of the (1) PIM and (2) nanoparticle-incorporated MM-PIM.

The DSC was conducted to analyse the chemical reactions and state changes of the membranes manufactured in the present study, such as glass transition (T_g_), crystallisation (T_c_), and melting (T_m_). Figure 5 demonstrates the DSC thermograms of the PIM and MM-PIM samples. The data were compared to determine the effects of the MWCNT nanoparticle loads on the chemical reactions and phase changes of the membranes.

The T_g_ values of the MM-PIM samples were nearly 9.5 °C greater than those of the PIM samples. The increment in the MM-PIM glass point revealed an improvement in the thermal stability of the membrane due to the presence of covalent bonding between the carbon nanotubes with the membrane polymer chains. The covalent bonds subsided the free volume and motility of the polymer chains, increasing the T_g_ values of the membranes [59,60].

The MM-PIMs demonstrated another apparent curve at the cold crystallisation temperature of 252.16 °C, exhibiting a smaller exothermic peak than the PIMs. The observation denoted that the carbon nanotubes induced a faster solidification rate of the MM-PIMs to the crystallisation phase. Furthermore, at 573.49 °C, a decomposition phase was observed on the PIM thermogram, whereas a minor endothermic peak was observed at a similar point in the MM-PIMs. The findings implied that the carbon nanotubes could prevent membrane mass degradation. Accordingly, the results affirmed that the incorporation of carbon nanotubes in PIMs could promote thermal stability and intermolecular interaction between the nanomaterials and different constituents in the membrane [61].

### 3.5. The IEC Study

**Figure 6:**
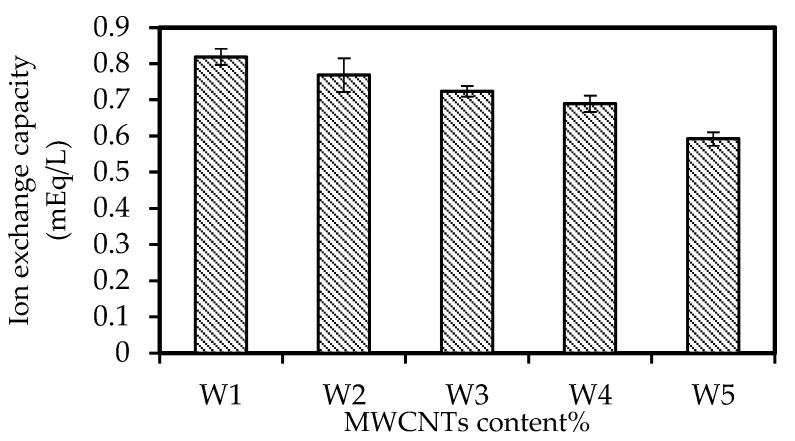
The IEC of the W1, W2, W3, W4, and W5 samples.

Figure 6 illustrates the IEC values of the W1, W2, W3, W4, and W5 samples. The data reveal that higher concentrations of nanomaterials lowered the IEC of the membrane from 0.819 to 0.592 mEq/L. The observation might be ascribed to the reduced access of potential of ion exchange groups into the membrane matrix due to increased particles invading the area surrounding the resin particles [53]. Furthermore, the larger inclusion concentration of nanomaterials in the doped solution caused more functional groups in the membrane matrix to be enclosed and detached by the CNT particles. Consequently, the accessibility of ion exchange was limited, which significantly affected the ion conductivity of the PIMs [52].

### 3.6. The EIS Analysis

**Figure 7:**
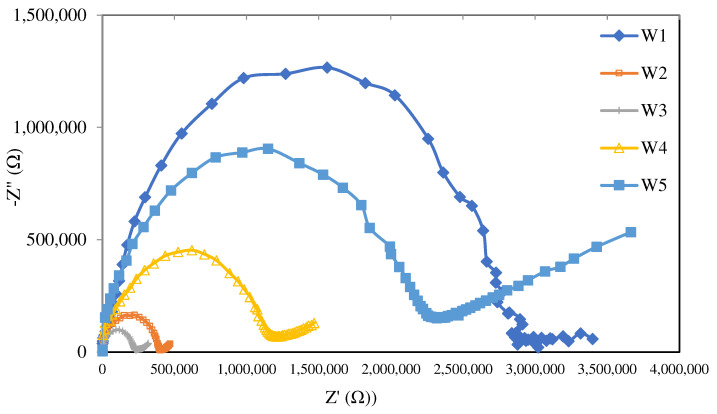
The Nyquist plots of the membrane samples at different MWCNT content amounts with an open-circuit voltage between 10 μHz and 10 MHz.

The EIS analysis was conducted to identify the effects of different nanoparticle loads on the conductivity profile of the membrane produced in the present study. Figure 7 presents the Nyquist plot of the PIM and MM-PIM samples at different MWCNT loads. Ideally, a Nyquist plot forms a semicircular line that describes a parallel linkage of a capacitor (static polymer chain) and a resistor (active ions inside polymer matrix) [62,63].

The impedance of the Nyquist plot in Figure 7 and the ion conductivity values in Table 4 exhibit apparent differences in resistance at different carbon nanotube content. The W3 membrane profile with 1.0% MWCNT content demonstrated the smallest impedance with the highest ion conductivity of 7.86 × 10^−^^8^ S cm^−1^. Ion conductivity increased with higher carbon nanotube content. The findings indicated that adding carbon nanotubes to the polymer matrix resulted in a gradual increase in ion conductivity from 2.47 × 10^−8^ to 7.86 × 10^−8^ S cm^−1^. The increment was likely due to improved electrostatic interactions between the particles in the matrix [43].

Higher membrane resistance was produced when the amount of MWCNTs reached over 1.0 wt%, resulting in the lower ion conductivity of the membrane. The results were in line with [64], stating that denser structures were formed on the membrane with increasing concentrations, which impeded the ion exchange sites and limited the ion transport pathways. Furthermore, the additive particles largely employed ionic paths in the membrane matrix and rejected ion transfer due to restricted channels, making MWCNTs a propitious nanoparticle for addition in the PIM.

### 3.7. Performance Studies of the Fabricated PIMs

#### 3.7.1. Optimisation of the Nanoparticle Compositions

Varying percentages of nanoparticles were doped in PIMs of similar formulations, which were 0.5, 1.0, 1.5, and 2.0% *w*/*w* MWCNTs, to evaluate the performance of the MM-PIMs in removing 10 mg/L zinc nitrate for five hours. The evaluation was performed to select the optimum nanoparticle composition that produced elevated absorption and uniform membranes. The mechanism involved in the target analytes’ transport was facilitated mass transport, where the extraction and back-extraction processes transpired simultaneously. The membrane transport process could be represented by Equations (11) and (12). The equations include different components, such as Zn^2+^, B2EHP, and neutral ion-pair complexes [65].

At the boundary layer or feeding phase:

Zn^2+^ + 3/2[RH]2(org) ^+^ → [ZnR2.HR](org) + 2H^+^ (11)

At the boundary layer or receiving phase:

[ZnR2.HR](org) + 2H^+^ → Zn^2+^ + 3/2[RH]2(org) (12)

where [RH]2 is the carrier, B2EHP and Zn(II) are the metal ions, and [ZnR2.HR](org) is the neutral ion-pair complex.

**Figure 8:**
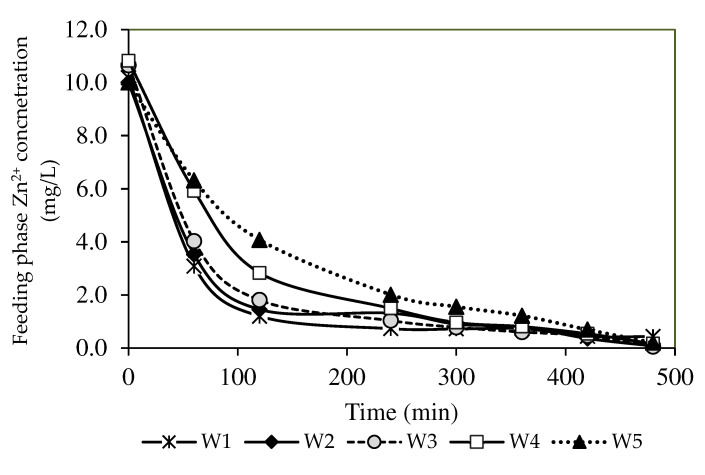
The percentages of Zn^2+^ removal of the PIM and MM-PIM samples in the feeding phase.

The role of MWCNTs is not described in the transport process in the abovementioned equations. Nonetheless, the effects of incorporating carbon nanotubes into the membranes could be identified from their presence in the membrane matrix. Figure 8 presents the concentration of Zn^2+^ removal in the feeding phase. The metal ion transport process across the applied fabricated PIM initially transpired via the removal of metal ions from the source phase, which then facilitated the diffusion of metal ions across the applied membrane with carrier binding. Finally, the metals were discharged into the receiving solution. The mechanisms for the extraction and back-extraction of the metal ions in Equations (11) and (12) were related to the carrier B2EHP, the cation exchanger in the zinc nitrate solution (Zn^2+^).

A PIM incorporated with an extracting agent and a nanoparticle could be employed as a sorbent in transporting arsenate and phosphate, which could occur without a receiving phase, considering that a similar anion-exchange mechanism is utilised to remove the target anions [4]. Consequently, an extracting agent significantly contributed to the transportation of cations from the source phase to the membrane. The agent also formed cation–carrier complex bonds to diffuse across the membrane, before separating from the complex bond to remove the metal ions into the receiving phase. On the other hand, the MWCNTs played a considerable role in providing strong membrane matrix mechanical stability to maintain membrane strength during the extraction processes.

The inclusion of nanoparticles in the membrane could also effectively promote the removal of metal ions. A study by [1] indicated that MWCNTs could be applied as Zn^2+^ sorbents (purified carbon nanotubes (CNT)) due to their huge surface area, rapid adsorption dynamics, and great adsorption capacity. For this reason, it is worth noting that the MWCNTs could work with the carrier to optimise the extraction of target ions into the receiving phase, hence generating higher metal ion ion exchange with the carriers across the PIMs.

**Figure 9:**
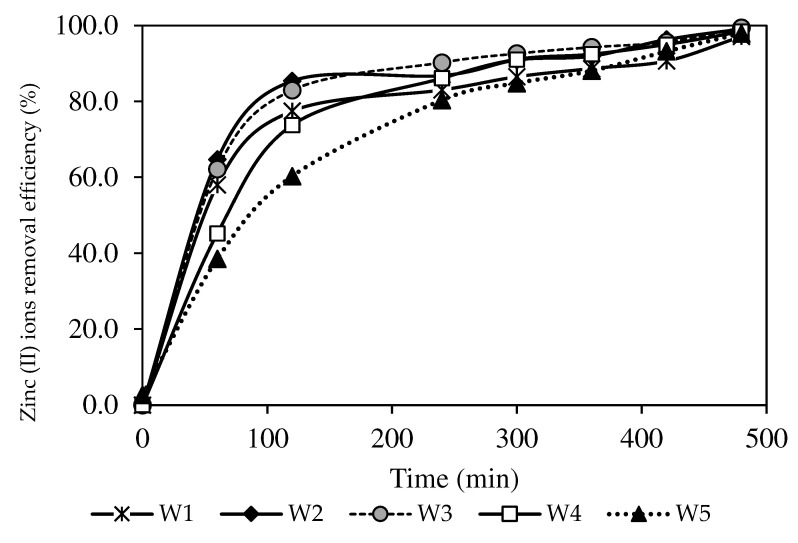
The Zn^2+^ removal efficiency percentages of the PIM and MM-PIM samples.

A descending trend of the Zn^2+^ concentration in the feeding phase was observed in the present study (see Figure 8). The results also demonstrated a greater capacity of Zn^2+^ removal, as evidenced by the increased Zn^2+^ removal efficiency in Figure 9. All fabricated PIMs with increasing MWCNT loads exhibited excellent removal performance of Zn^2+^ concentrations below 0.5 mg/L for seven hours (Figure 8). The W3 membrane at 99.44% obtained the highest removal efficiency with a 1.0% MWCNT load than the W1 sample that was not incorporated with nanoparticles. The incorporation of carbon nanotubes into PIMs facilitated the transport of Zn^2+^ through the membrane. A report by [4] stated that the MWCNT content in the membrane matrix could boost the complexation rate of ion-pair formation on the membrane surface, resulting in a higher concentration gradient of the complex in the membrane matrix. The findings signified that a larger [ZnR2.HR](org) complex formed in the PIMs, which imposed a greater transport driving force before the membrane reached saturation [66].

The W4 and W5 membranes with MWCNT loads of over 1.0% demonstrated a lower diffusion rate than the W1, W2, and W3 membranes before four hours. The increased concentration of nanoparticle inclusion linearly increased the membrane density, leading to a lower [ZnR2.HR](org) complex in the membrane [67]. Furthermore, water uptake characterisation in Table 4 describes the MWCNT content above 1.0% as hydrophobic, since the bonding region between metal ions and the membrane surface is smaller. Accordingly, the removal of Zn^2+^ was favourable through the W1, W2, and W3 membranes.

**Figure 10:**
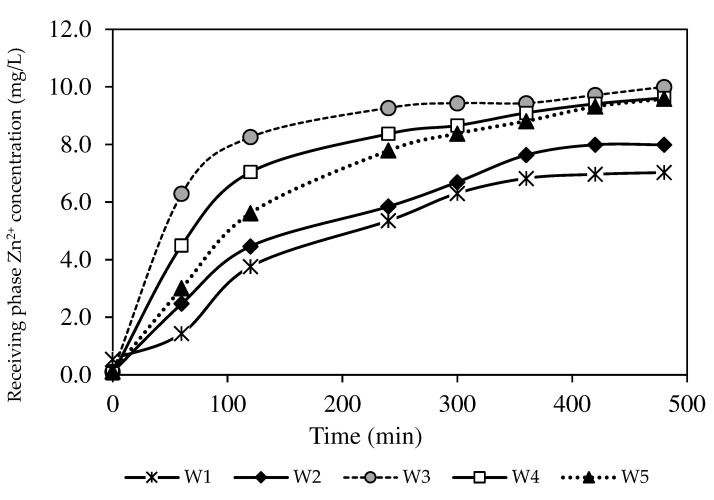
The Zn^2+^ removal percentages of the PIM and MM-PIM samples in the receiving phase.

The concentration of Zn^2+^ collected in the receiving phase is displayed in Figure 10. The line plot in Figure 10 exhibits an ascending trend of the Zn^2+^ concentration in the receiving phase, demonstrating that extraction and back-extraction processes occurred concurrently in reverse phases of the feeding and receiving phases. The W3 membrane had the highest Zn^2+^ concentration, close to 10 mg/L, whereas the W1 membrane possessed the lowest concentration of Zn^2+^ at 7.03 mg/L. The data implied that the back-extraction process was less effective for the PIMs without nanoparticles.

The transported Zn^2+^ recovered was less compared to the extraction process in the feeding phase. The observation was likely due to the poor dissociation rate of the ion-pair complex at the PIM-receiving interphase to remove target ions into the receiving solution. The same results were obtained from the W4 and W5 membranes that contained carbon nanotubes over 1.0%. A lower de-complexation of ion-pair formation with increased MWCNT load possibly produced denser membranes.

**Figure 11:**
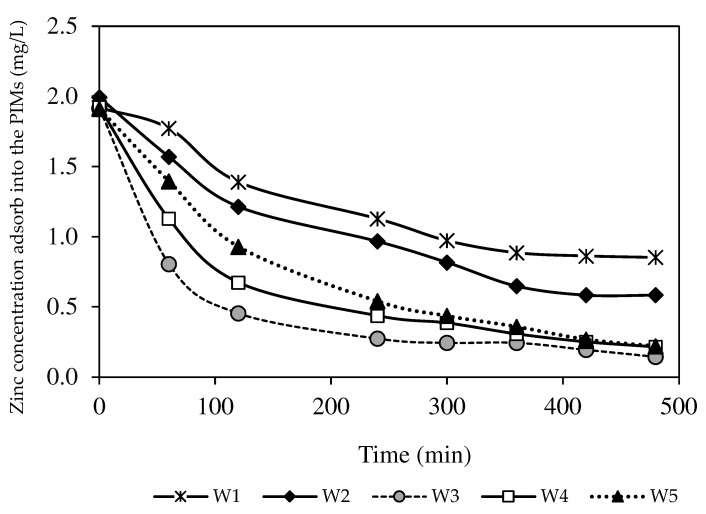
The removed Zn^2+^ percentages absorbed into the PIM and MM-PIM membranes.

The inferior efficiency of the PIMs without nanoparticles was proven from the concentration of Zn^2+^ absorbed in the membrane (see Figure 11). Although the W1 membrane imposed the excellent transport of Zn^2+^ from the feeding phase, the amount of Zn^2+^ trapped and stored in the membrane due to lower dissociation of ion-pair formation was higher than the number of target ions extracted back into the receiving phase. A similar outcome was reported by [47], where, during the filtration process, the bare membrane permitted foulants to assimilate on the membrane surface or precipitate inside the membrane pores. Resultantly, there was reduced membrane hydrophilicity to extract target analytes across the membrane [39,40]. Another factor was the high inclusion of nanoparticles that could occupy the spaces in the membrane rather than target ions, resulting in ionic exchange site blockage and an ion transport pathway shortage [23,49]. Consequently, the W3 membrane with 1.0% MWCNT load was chosen as the optimised MM-PIM for further studies, since it provided better extraction and back-extraction performance in transporting and removing Zn^2+^.

#### 3.7.2. Optimisation of Essential Parameters for Optimal Membrane Performance

##### The Effects of Different Initial Concentrations of Source Phase

**Figure 12:**
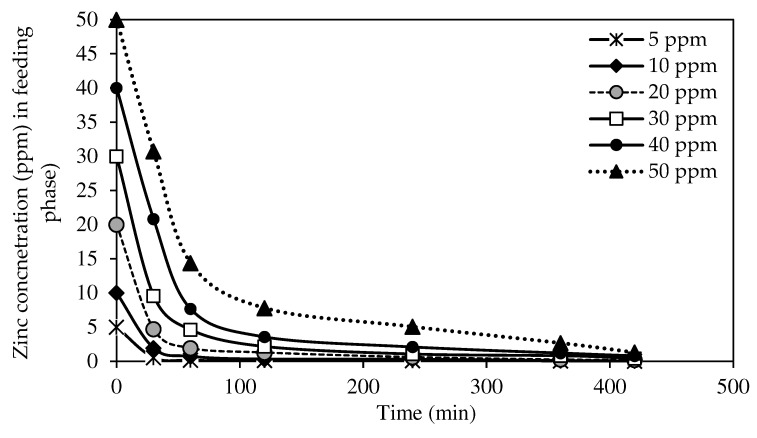
The effects of initial Zn^2+^ concentration on metal ion transport of the W3 membrane.

**Figure 13:**
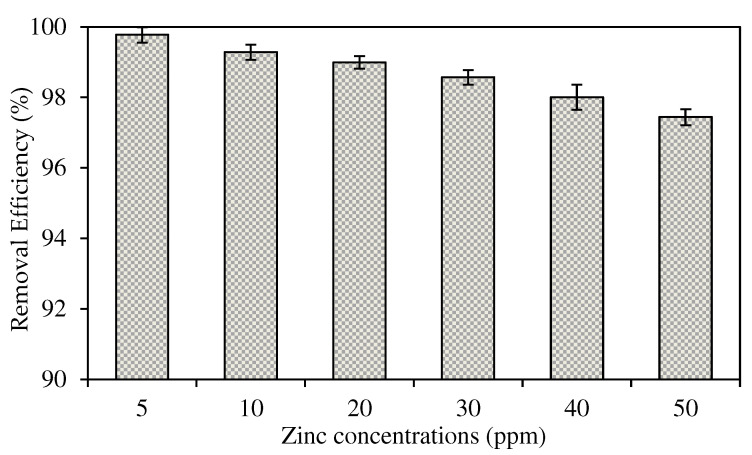
The Zn^2+^ removal efficiency percentage of the W3 membrane.

The selected W3 MM-PIM membrane was employed to investigate the effects of the initial concentration of zinc nitrate on the membrane efficiency to remove the Zn^2+^. Figure 12 exhibits Zn^2+^ concentrations at different initial feed concentrations at the feeding phase. Figure 13 presents the removal efficiency of the W3 membrane. The concentration of the feeding phase was varied within the 5 to 50 ppm range.

Based on the results, the optimum initial feed concentration value was 5 mg/L with 99.78% removal efficiency. The maximum Zn^2+^ removal was reached quicker than higher initial feed concentrations. The rate of ion-pair complex transport was the fastest in low initial feed concentrations in removing metal ions across the membrane. Generally, the percentages of Zn^2+^ removal in different concentrations of the feeding phase were >97%. Nevertheless, the removal efficiency percentage decreased as the initial feed concentration increased. The observations were likely due to the carrier saturation on the surface of the membrane, which reduced the effective transportation membrane area.

The transport of Zn^2+^ was faster in initial feed concentrations of <50 ppm as the maximum removal was reached in less than four hours. The data demonstrated that integrating MWCNTs into the PIM assisted in accelerating the transport of Zn^2+^ at different feed concentrations. Despite the fixed carrier composition in the membrane, the nanotubes incorporated had sorbent properties that enlarged the effective surface area on the membrane for the binding of target ions before membrane saturation developed [4].

##### The Effects of Different Receiving Agent Concentrations

**Figure 14:**
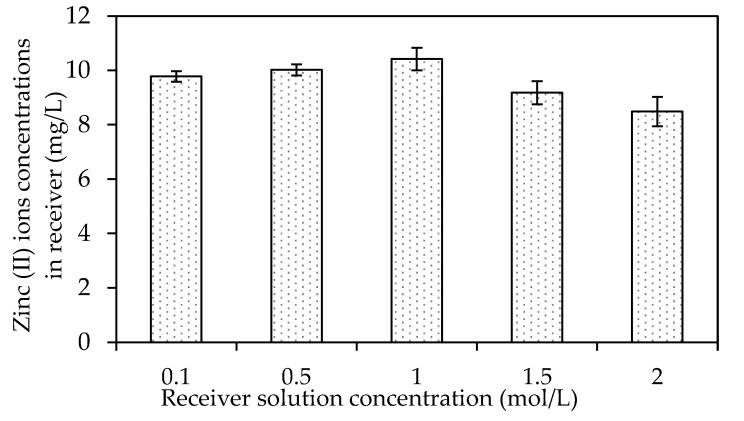
The effects of different receiver solution concentrations on the concentration of Zn^2+^ in the receiving phase.

The effects of different receiving agent concentrations were assessed on the selected W3 membrane. The evaluation investigated the optimum acidity condition in the receiver solution to maximise the back-extraction process in the receiving phase. Figure 14 presents the effects of different receiver solutions on the concentration of Zn^2+^ in the receiving phase. In the present study, the feed solution was maintained at 100 ppm and pH 2. The pH was maintained at precisely two to prevent the carrier from becoming unbalanced, since the pKa of B2EHP is 3.5, and the possibility of carrier leaching increases when the pH is ionised at higher than 3 [7,30]. The PVC was more stable in the membrane matrix since the base polymer was less prone to producing dehydrochlorination in a more acidic solution.

According to [68], nitric acid is an excellent receiving solution as it allows vast metal ion uptake compared to other acidic solutions. Consequently, the solution was utilised as the receiving phase within the concentration range of 0.1–2.0 mol/L in the transport experiment. The metal ion transfer process from the feeding to receiving phase was counter-coupled to the transport of H^+^ in the reverse course. As the gradient of the hydrogen concentration across the membrane increased, the driving force of the target ions’ transport also increased.

Based on Figure 14, an increasing trend of Zn^2+^ removal efficiency was observed with the increasing concentration of the receiver solution, 0.1 to 1.0 M nitric acid. The data suggested that more protonation occurred in the receiver solution with the increasing concentration of the receiving phase and escalated the driving force to facilitate the dissociation of the ion-pair complex at the surface of the membrane to the receiver phase. Accordingly, a higher receiver solution concentration might significantly improve the back-extraction of Zn^2+^ into the receiving phase.

An apparent declination was demonstrated in the Zn^2+^ concentrations desorbed after the receiver solution increased from 1.5 to 2 M. Research by [68] found that nitric acid as the receiver concentration (>1.0 mol/L) did not significantly affect the reduction of metal ions, demonstrated by the lack of difference in the statistical data of the experiments. An acid concentration of <1.0 mol/L is more convenient as the receiving solution as it is more desirable considering the cost and safety [68,69]. Consequently, the present study selected the receiver concentration of 1.0 M of HNO_3_ in removing Zn^2+^ with the MM-PIM. The source solution had a Zn^2+^ concentration of 10 ppm concentration at pH 2 and a transport time of five hours.

**Figure 15:**
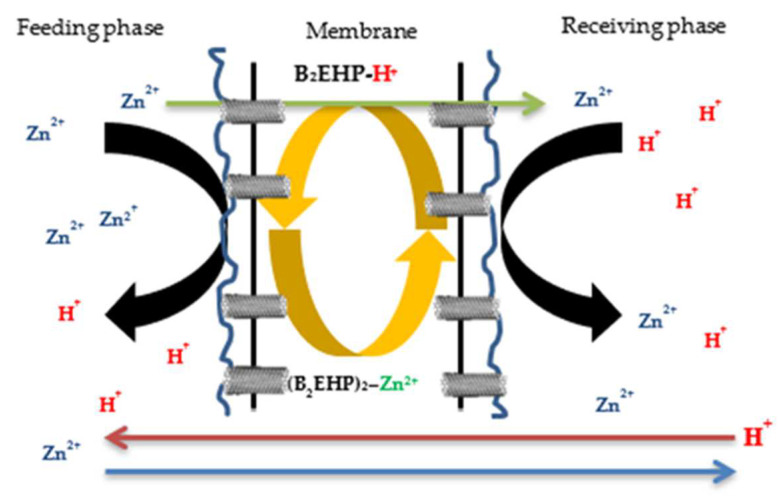
Illustrations of zinc (II) ion extraction process.

The mechanisms of zinc (II) ion removal can be explained by Figure 15, which shows the illustrations of zinc (II) ion removal by binding to the carrier, B2EHP, and with the support of nanoparticles, namely MWCNTs. According to [69], the extraction process of the target cations using PIM extraction involved three succeeding steps. Firstly, the carriers underwent proton ionisation and protons were released into the feeding phase, producing a negatively charged compound. Then, the cations were bonded to the carrier, resulting in the formation of weak Van der Waals and hydrogen bonds at the carrier’s active sites and, consequently, an ion-pair complex was formed. Then, the cations were transported over the surface of the membrane into the receiving phase and, lastly, the cation was released into the receiving solution once the ion-pair complex dissociated through the back-extraction process. In return, the cation from the receiving phase was replaced by the hydrogen ion from the carrier, and the carrier was returned to the feeding solution, where the extraction process was repeated.

The application of carbon nanotubes for the fabrication of membranes by directly adding them to the membrane casting solution appears to be a viable strategy. When a membrane is fabricated via the phase inversion method, CNTs inserted into the casting dope significantly modify the membrane’s porosity properties, thus improving membrane performance [70]. Research by [71] reported that ionic diffusion and ‘gatekeeper’ activity are the two main methods of mass transfer across CNTs. The word ‘gatekeeper’ refers to a chemical layer positioned at the pore entrance that selectively enables substances to move into and through the membrane’s pores. Therefore, through the adsorption process, the cations in the feeding phase likely underwent fast transport along the CNTs’ surface to diffuse into the receiving phase.

### 3.8. Kinetic Studies

#### 3.8.1. The Effects of Different Initial Feed Phase Concentrations on Zn^2+^ Removal with the W3 Membrane

**Figure 16:**
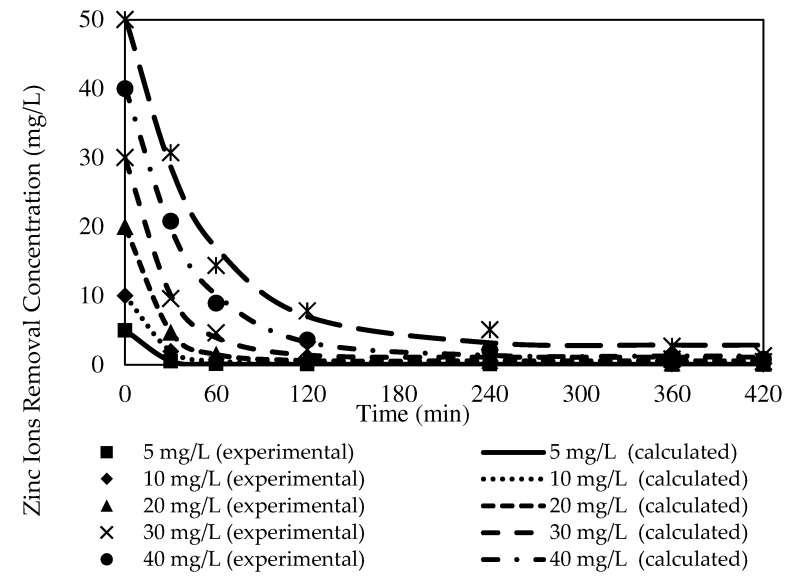
Fitting of the experimental data to the PFO model describing Zn^2+^ removal by the W3 membrane over time at different initial source phase concentrations.

**Figure 17:**
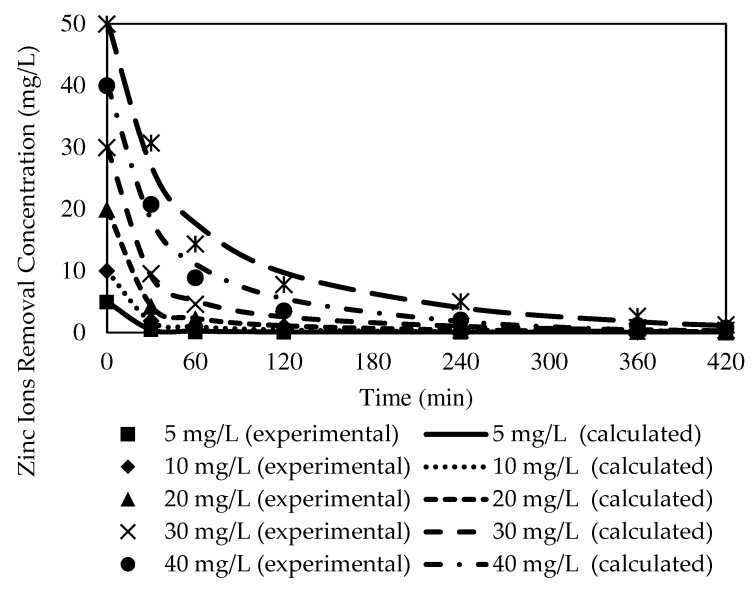
Fitting of the experimental data to the PSO model describing Zn^2+^ removal by the W3 membrane over time at different initial source phase concentrations.

The kinetic data obtained from the numerical calculations of kinetic models for each initial concentration of feed phase with the W3 membrane are presented in Table 5 and Table 6. The numerical calculations included the theoretical equilibrium concentration, C_e_, rate constants, K_1_, theoretical initial concentration, C_0_, correlation coefficient, R^2^, and variance. The fitted plots of the experimental and theoretical data for the PFO and PSO models in removing Zn^2+^ are illustrated in Figure 16 and Figure 17.

Based on Table 5, as the initial feed concentration increased, the K_1_ value decreased from 0.0788 to 0.0203. The highest reaction rate for Zn^2+^ removal was obtained at 5 mg/L at 0.0788, whereas 50 mg/L exhibited the lowest rate of reaction at 0.0203. Similarly, K_2_ values of 0.0599 to 0.0005 demonstrated 5 mg/L as the highest rate of reaction at 0.0599, while the lowest rate of reaction was acquired at 50 mg/L at 0.0005. Despite the transverse trends between the initial feed concentration and rate of reactions exhibited by the numerical data, both kinetic models presented significantly low C_e_ compared to the C_0_ of the respective models.

A study by [70] indicated that higher potential for overall resistance existed in the membrane due to the emergence of the fouling layer on the membrane surface over time with an increasing feeding solution concentration. The layer interrupted the mass transfer of Zn^2+^ through the membrane and lowered the membrane performance. Accordingly, it could be assumed that the antifouling property of the carbon nanotubes incorporated into the PIMs manufactured in the present study restrained the fouling production and sustained the hydrophilicity of the membrane. A greater dissociated ion-pair complex with hydrogen ion (H^+^) bonding at the membrane and receiver interphase permitted the removal of Zn^2+^ into the receiving phase [47].

A comparison of the correlation coefficient values of the models in Table 5 and Table 6 revealed that the values of R^2^ presented by the PFO model were better fitted to the experimental data than those of the PSO model. The PFO model demonstrated significantly higher R^2^ values, 0.9996, 0.9990, 0.9979, 0.9972, 0.9974, and 0.9912, according to the initial feed solution concentrations. Furthermore, most of the calculated variances in the PFO model (see Figure 5) were noticeably lower than the variances of the PSO model. The observations signified that the PFO could depict the kinetic mechanisms of Zn^2+^ removal better than the PSO model.

#### 3.8.2. The Effects of Different Receiving Agent Concentrations with the W3 Membrane

**Figure 18:**
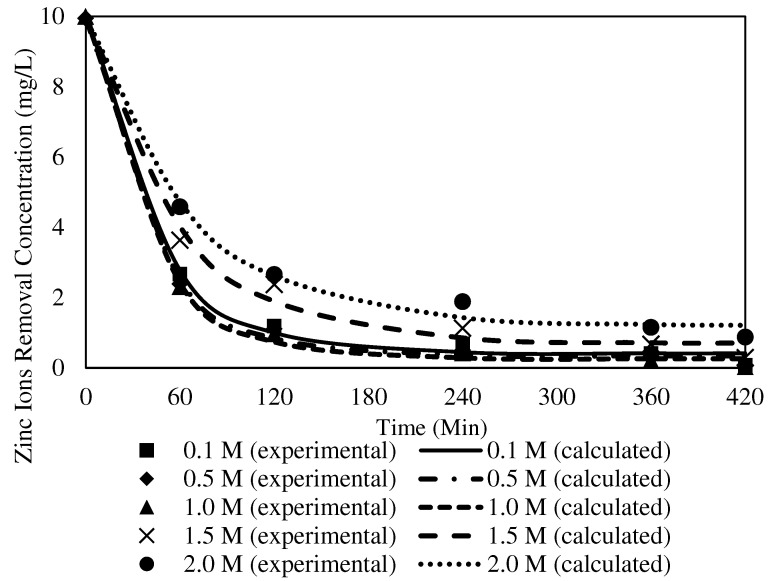
Fitting of the experimental data to the PFO model describing Zn^2+^ removal with the W3 membrane over time at different receiving agent concentrations.

**Figure 19:**
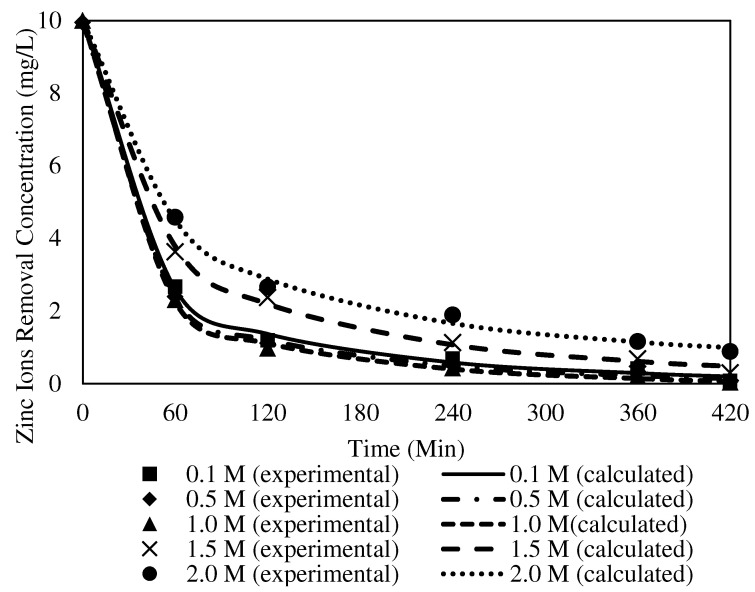
Fitting of the experimental data to the PSO model describing Zn^2+^ removal with the W3 membrane over time at different receiving agent concentrations.

The theoretical C_e_, K_1_, C_0_, R_2_, and variances of the PFO and PSO models’ experimental parameters at different receiving agent concentrations with the W3 membrane are tabulated in Table 7 and Table 8. Figure 18 and Figure 19 present the tabulated results of the transport mechanisms, demonstrating the fitted plots of the experimental and calculated data for the concentration of Zn^2+^ removal versus time for both kinetic models.

The R^2^ values of the PFO model for Zn^2+^ removal at different concentrations of receiving agent were in the range of 0.9960 to 0.9991. The K_1_ values of the PFO model indicated that the rate of reaction increased at higher receiver concentrations, up to 1.0 mg/L. Accordingly, 1.0 mg/L delivered the highest rate of reaction based on the PFO model at 0.0251. The incorporated MWCNTs in the polymeric membrane were responsible for promoting the transport process as a sorbent to enhance the transfer of metal ions through the W3 membrane, mainly to conduct the ion exchange between the ion-pair complex and higher concentrations of H^+^ in the receiving phase at the membrane and receiver interphase. The findings were in line with [72], as the particles of carbon nanotubes possess adsorption properties that develop greater interactions with target ions to the surface of the membrane. The transport of ion-pair complexes was facilitated to separate the metal ions across the membrane. Accordingly, it is reasonable to suggest that the nanoparticles stimulate a better outcome in removing Zn^2+^.

When the receiver concentrations applied in the transport experiments were >1.0 mg/L, the K_1_ values decreased, with a slower rate of Zn^2+^ removal. A receiver concentration of 2.0 mg/L demonstrated the lowest reaction rate at 0.0152. The undesirable outcomes were most likely due to proton saturation in the receiving phase, which reduced the accessibility of the metal ions to permeate through the sorbent in the membrane matrix [23,73]. Resultantly, the percentage of Zn^2+^ removed was reduced.

Under similar parameter conditions, the R^2^ of the PSO model ranged from 0.9949 to 0.9986. The highest K^2^ value was obtained from 1.0 mg/L at 0.0046, whereas the lowest K^2^ value appeared at 2.0 mg/L with 0.0020. The R^2^ values of the PFO model were significantly higher than those of the PSO model. The finding implied that the PFO model best fitted the numerical data for different receiver concentrations. A similar observation on the physisorption mechanism was reported by [74], where weak van der Waals interactions among the particles in the membrane matrix existed. Consequently, the kinetic mechanism of Zn^2+^ removal at different receiving agent concentrations was presumed to obey the PFO that occurred in the physisorption mechanism.

### 3.9. Permeation Study on PIMs with Optimised Parameters

#### 3.9.1. The Effects of Different Initial Concentrations

Table 9 summarises the effects of the initial feed concentrations on the permeability and flux of Zn^2+^ removal by W3. The initial zinc (II) concentrations in the feeding phase varied within the 5 and 50 mg/L range. The flux values increased from 0.0836 to 0.2462 mol m^−^^2^ s^−^^1^ as the concentration of receiver solution was increased. Conversely, the permeability values decreased with inclining Zn^2+^ concentrations from 0.0167 to 0.0043 m s^−^^1^. Nevertheless, initial feed concentrations of >30 mg/L exhibited a noticeable decrement in membrane flux, 0.2462 and 0.2112 mol m^−^^2^ s^−^^1^, respectively, at concentrations of 40 and 50 mg/L.

A similar outcome was revealed by [75], demonstrating the potential reduction in cation flux with higher initial concentrations due to the accumulation of molecules deposited on the surface of the membrane, leading to membrane fouling. In the current study, it was likely due to ion-pair complexes that had fully occupied the membrane pores, aside from carrier or nanoparticle build-up on the surface of the membrane. The severely reduced effective membrane surface area and the retention of separate components on the increased flow side produced reduced flux.

#### 3.9.2. The Effects of Different Feed Phase Initial Concentrations with the W3 Membrane

Table 10 presents the values of permeability and flux obtained from the Zn^2+^ removal process utilising the W3 membrane at different receiving phase concentrations. The receiver concentrations ranged from 0.1 to 2.0 mol L^−1^ and the pH was maintained at 2 in all receiving phase solutions.

The permeability coefficient and flux gradually increased with increasing receiver concentrations (0.1 to 1.0 M) at 0.0049 to 0.0053 m s^−1^ and 0.0495 to 0.0532 mol m^−2^ s^−1^, respectively. The observations could be attributable to the effective diffusion of target ions across the membrane matrix through intermolecular forces with the MWCNT sorbent at the PIM–receiver interface, which subsequently promoted the driving force of the higher H^+^ concentration in the receiving phase to transfer into the feeding phase. According to [33], the surface of the MWCNT particles is composed of negatively charged particles that often electrostatically attract almost every positively charged metal species to form ion-pair complexes. For this reason, the adsorption of metal ions through PIMs integrated with carbon nanotubes presumably sped up and eased the removal process.

As the concentration of the receiver solution was increased beyond 1.0 M, the permeability and flux values declined. The reason was the limited available membrane surface top8ermit the diffusion of H^+^ through the H^+^ ion concentration gradient from the receiving to the feeding phase due to the retention of molecules in the nanotube’s columns. Accordingly, a higher receiver solution concentration might result in the deceleration of ion exchange to transport Zn^2+^ through the PIM and receiver interface [76]. Consequently, a receiving solution of 1.0 M nitric acid was preferable as the receiving agent to remove Zn^2+^ across the W3 membrane.

## 4. Conclusions

The incorporation of MWCNT nanoparticles into the cation exchange PIM was performed and characterised successfully. The PIM with 1.0% MWCNT nanoparticles exhibited excellent Zn^2+^ removal performance compared to other MM-PIMs. Furthermore, the embedded carbon nanotubes improved the PIM’s characteristics, such as a coarser surface, highly hydrophilic nature, and higher conductivity, to alleviate the removal process. The performance of the carrier-mediated PIM was facilitated by the MWCNT content. Moreover, a 5 mg/L initial feed concentration presented the highest Zn^2+^ removal, while the optimal receiving agent concentration was 1.0 mg/L of nitric acid. The removal process was established through the physisorption mechanism as the kinetic study tended to favour the PFO model. Accordingly, the W3 membrane was described as having the best fit to all PFO model parameters. Permeability and flux investigations were also conducted by employing the obtained kinetic data of the PFO model.

## Figures and Tables

**Table 1:** Examples of different formulations of PIMs and their applications.

Base Polymer	Plasticiser	Solvent	Carrier	Target Analytes	Removal Efficiency	References
CTA	2-NPPE	TBP	Aliquat 336	Co(II)	100%	[11]
CTA	NPOE	DCM	Cyphos IL 104	Fe(III)	89.6%	[12]
CTA	PBAT	-	Aliquat 336	Cr(IV)	>99.0%	[10]
CTA	2-NPOE	-	Calix [4]arene	Cr(IV)	97.69%	[13]
CTA	*o*-NPPE	-	1-decyl-imidazole	Zn(II)	92.5%	[14]
PVC	-	THF	Alqiuat 336	Thiocyanate	99%	[6]
PVC	DOP	THF	B2EHPA	Malachite Green (MG)	96%	[15]
PVC	DOP	THF	Aliquat 336	Cu(II)	99.9%	[16]
PVC	-	THF	Aliquat 336	Cd(II)	95.0%	[17]
PVC	ADO	-	3-propylacetylacetone	Zn(II)	93.0%	[18]
PVC	ILPs	-	Cyphos IL 104	Cr(VI)	99.5%	[19]

**Table 2:** Formulations of the MM-PIMs employed in the current study.

Batch Code	Membrane	PVC (wt%)	B2EHP (wt%)	DDOP (wt%)	THF (wt%)	MWCNT (wt%)
0030	W1	18	30	1	51.0	0.0
0530	W2	18	30	1	51.0	0.5
1030	W3	18	30	1	51.0	1.0
1530	W4	18	30	1	51.0	1.5
2030	W5	18	30	1	51.0	2.0

**Table 3:** The wavenumbers of significant peaks observed in the FTIR spectra of the W1 (PIM) and W3 (MM-PIM) samples.

Material	Wavenumber (cm^−1^)	Type of Molecular Vibration
Before Zn^2+^ removal		
W1 (PVC 18%:B2EHP 30%:DOP %:THF 51%)-PIM	2958.39–2929.20 2859.91 1716.99 1458.98 1227.90 1018.50 884.73–727.28 695.63–610.40	C_sp3_-H C_sp2_-H C=O C–C P=O/O–C P–O C–H ‘oop’ C–Cl
W3 (MWCNT 1%:PVC 18%:B2EHP 30%:DOP %:THF 51%)-MM-PIM	2958.30–2929.44 2860.00 1716.84 1458.70 1228.68 1018.20 884.11–727.00 692.40–611.34	C_sp3_-H C_sp2_-H C=O C–C P=O/O–C P–O C–H ‘oop’ C–Cl
After Zn^2+^ removal		
W1 (PVC 18%:B2EHP 30%:DOP %:THF 51%)-PIM	2960.47–2929.89 2860.20 1716.75 1463.71 1258.63 1012.36 865.59–794.42 697.12–611.17	C_sp3_-H C_sp2_-H C=O C–C P=O/O–C P–O C–H ‘oop’ C–Cl
W3 (MWCNT 1%:PVC 18%:B2EHP 30%:DOP %:THF 51%)-MM-PIM	2960.34–2929.94 2860.15 1717.18 1425.55 1258.77 1012.86 868.65–795.90 695.51–611.45	C_sp3_-H C_sp2_-H C=O C–C P=O/O–C P–O C–H ‘oop’ C–Cl

**Table 4:** The CA, water uptake, porosity, and thickness of the PIM and MM-PIM samples.

Membrane Sample	CA (°)	Water Uptake, *U* (%)	Porosity, ε(%)	Thickness (mm)	Ion Conductivity (mS cm^−1^)
W1	52.5	50.21	32.98	0.06 ± 0.01	4.62 × 10^−9^
W2	51.6	47.26	28.77	0.08 ± 0.01	2.47 × 10^−8^
W3	45.8	45.51	26.35	0.08 ± 0.01	7.86 × 10^−8^
W4	43.5	41.04	20.12	0.09 ± 0.01	7.26 × 10^−8^
W5	43.9	39.25	19.21	0.09 ± 0.01	8.41 × 10^−9^

**Table 5:** The parameter values of the PFO models obtained by numerical calculation for the removal process by the W3 membrane with different initial concentrations.

Initial Concentration (mg/L)	Ce	K_1_	C_0_	R^2^	Variance
5	0.0678	0.0788	4.9997	0.9996	0.0018
10	0.2167	0.0570	9.9942	0.9990	0.0195
20	0.5704	0.0512	19.9939	0.9979	0.1652
30	1.1074	0.0388	29.9100	0.9972	0.4803
40	1.2733	0.0247	40.2138	0.9974	0.8143
50	2.8219	0.0203	50.4390	0.9912	4.2980

**Table 6:** Parameter values of the PSO models obtained by numerical calculation for the removal process by the W3 membrane with different initial concentrations.

Initial Concentration (mg/L)	C_e_	K_1_	C_0_	R^2^	Variance
5	0.0231	0.0599	5.0002	0.9993	0.0037
10	0.1270	0.0142	10.0025	0.9987	0.0256
20	0.2626	0.0057	20.0089	0.9973	0.2131
30	0.6751	0.0023	30.0244	0.9989	0.1928
40	2.7380	0.0008	40.3296	0.9869	4.1776
50	3.4251	0.0005	50.6057	0.9841	7.7936

**Table 7:** The calculated numerical parameter values of the PFO models for the W3 membrane removal process with different receiving agent concentrations.

Receiving Agent Concentration (mg/L)	Ce	K_1_	C_0_	R^2^	Variance
0.1	0.2107	0.0233	10.0054	0.9969	0.0748
0.5	0.3413	0.0246	9.9347	0.9983	0.0656
1.0	0.6600	0.0251	9.9877	0.9991	0.0464
1.5	0.4957	0.0171	9.9660	0.9974	0.2127
2.0	0.1983	0.0152	9.9716	0.9960	0.1174

**Table 8:** The calculated numerical parameter values of the PSO models for the W3 membrane removal process with different receiving agent concentrations.

Receiving Agent Concentration (mg/L)	C_e_	K_1_	C_0_	R^2^	Variance
0.1	0.3610	0.0040	10.0219	0.9949	0.0258
0.5	0.3625	0.0045	9.9510	0.9982	0.0103
1.0	0.4282	0.0046	10.0017	0.9986	0.0100
1.5	0.2438	0.0024	10.0297	0.9976	0.0307
2.0	0.1788	0.0020	10.0146	0.9979	0.0413

**Table 9:** The permeability and flux values of Zn^2+^ removal by the W3 membrane at different initial concentrations.

Initial Concentration (mg/L)	Permeability Coefficient (m s^−1^)	Flux(mol m^−2^ s^−1^)
5	0.0167	0.0836
10	0.0121	0.1209
20	0.0109	0.2172
30	0.0082	0.2462
40	0.0052	0.2127
50	0.0043	0.2112

**Table 10:** The permeability and flux values for Zn^2+^ removal by the W3 membrane at different receiving agent concentrations.

Receiving Agent Concentration (M)	Permeability Coefficient (m s^−1^)	Flux(mol m^−2^ s^−1^)
0.1	0.0049	0.0495
0.5	0.0052	0.0519
1.0	0.0053	0.0532
1.5	0.0036	0.0362
2.0	0.0032	0.0322

## Data Availability

The data presented in this study are openly available in the results and discussion section of this study.
